# Assessment on reticuloendotheliosis virus infection in specific-pathogen-free chickens based on detection of yolk antibody

**DOI:** 10.1371/journal.pone.0213978

**Published:** 2019-04-22

**Authors:** Yang Li, Tuanjie Wang, Lin Wang, Mingjun Sun, Zhizhong Cui, Shuang Chang, Yongping Wu, Xiaodong Zhang, Xiaohui Yu, Tao Sun, Peng Zhao

**Affiliations:** 1 China Animal Health and Epidemiology Center, Qingdao, China; 2 China Institute of Veterinary Drug Control, Beijing, China; 3 College of Veterinary Medicine, Shandong Agricultural University, Taian, China; 4 College of Animal Sciences and Technology, Zhejiang A&F University, Hangzhou, China; 5 Shandong Entry-exit Inspection and Quarantine Bureau, Qingdao, China; The University of Hong Kong, CHINA

## Abstract

Reticuloendotheliosis virus (REV) is the most frequent exogenous virus that contaminates attenuated vaccines. Therefore, it is extremely important to select REV-free specific-pathogen-free (SPF) chicken embryos. Generally, REV infection is assessed by detecting REV antibodies in SPF chickens. This present study seeks to evaluate REV infection by replacing serum antibody detection with yolk antibody detection. A cohort of 40 nineteen-week-old SPF chickens were artificially inoculated with REV, with 32 SPF chickens raised in another isolation environment served as a blank control. Eggs and serum from 23-week-old chickens were sampled, and yolks were diluted separately to ratios of 1:150, 1:200, 1:300 and 1:400, which were detected together with serum. We found that the yolk antibody detection findings at a dilution of 1:300 had the highest coincidence rate compared with that based on serum antibody measurements. At a dilution ratio of 1:300 for yolk antibody, 72 chickens were continuously observed for 10 weeks from 25- to 34-weeks-old. Our findings were based on serum antibody or yolk antibody detection, and the evaluation results were completely consistent. Therefore, all serum antibody-positive chickens were yolk antibody-positive, and vice versa. Accordingly, vaccine producers can estimate REV cleanliness in a poultry farm by sampling yolk antibody titers.

## Introduction

Avian reticuloendotheliosis virus (REV) is one of the most important pathogens that can cause avian tumors. Recently, epidemiological investigations showed that REV infection is very common in Chinese chickens, particularly in local poultry species [1–3]. As REV can be vertically transmitted through hatching eggs [4], if REV-contaminated eggs are used to produce attenuated vaccines, vaccines can be contaminated by REV, which represents one of the crucial ways to disseminate REV [5–7]. Recently in China, the use of REV-contaminated attenuated vaccines is considered to be an important cause of REV infection [8–10].

To overcome this problem, as the Ministry of Agriculture of China stipulated, all attenuated poultry vaccines must use SPF chickens as raw materials to produce attenuated vaccines, and all vaccine producers must confirm whether SPF chickens are infected by REV or not using sampled serum antibody detection. However, because of the specificity of housing standards in SPF poultry farms, others cannot freely enter a breeding area for sampling and detection. In this current study, we attempted to replace antibody detection in serum with antibody detection in egg yolks of SPF chickens.

## Results

### Determination of the optimal yolk dilution

Under the same conditions, we measured REV antibody titers in paired yolk and serum samples collected on the same day or one day before or after in 40 SPF chickens during the initial egg-laying stage when the chickens were 23 weeks old. Table 1 shows the “goodness of fit” between yolk antibody titers diluted to various concentrations and serum antibody titers at the required concentration. By comparison, we found that REV antibody detection in the yolk at a 1:300 dilution had the highest goodness of fit with serum antibody measurements, and reached 97.5%.

**Table 1:** Consistent yolk and serum antibody measurements with different dilutions of yolk.

Dilution of yolk	yolk antibody consistent well with serum antibody			yolk antibody is on the contrary to serum antibody		
	Yolk (positive) Sera (positive)	Yolk (negative) Sera (negative)	Total	Yolk (positive) Sera (negative)	Yolk (negative) Sera (positive)	Total
1:150	21	16	37	2	1	3
1:200	21	16	37	2	1	3
**1:300**	**21**	**18**	**39**	**0**	**1**	**1**
1:400	21	16	37	1	2	3

### Comparison of the goodness of fit for ALV-Ab antibody measurements in serum and yolk from SPF chickens of different ages

In 25–34-week-old chickens, serum and hatching eggs were sampled once per week, and a total of 720 serum samples and 720 yolk samples were collected from 40 SPF infected chickens and 32 SPF chickens without virus challenge. Table 2 showed that the yolk antibody findings were completely consistent with those based on serum antibody detection within 10 weeks, as the serum antibody-positive chickens were all yolk antibody-positive, and the serum antibody-negative chickens were all yolk antibody-negative. Additionally, 35 of 40 SPF chickens challenged with REV alone were always REV antibody-positive in the serum and yolk, while 4 were always REV antibody-negative. All 32 SPF chickens without virus challenge were always REV antibody-positive in the serum and yolk. The goodness of fit for serum antibody and yolk antibody detection reached 100%.

**Table 2:** Agreement of yolk and serum antibody measurements with different dilutions of yolk.

Weeks	yolk antibody consistent well with serum antibody			yolk antibody is on the contrary to serum antibody		
	Yolk (positive) Sera (positive)	Yolk (negative) Sera (negative)	Total	Yolk (positive) Sera (negative)	Yolk (negative) Sera (positive)	Total
25w	36	36	72	0	0	0
26 w	36	36	72	0	0	0
27 w	36	36	72	0	0	0
28 w	36	36	72	0	0	0
29 w	36	36	72	0	0	0
30 w	35	37	72	0	0	0
31 w	35	37	72	0	0	0
32 w	35	37	72	0	0	0
33 w	35	37	72	0	0	0
34 w	35	37	72	0	0	0
**Total**	355	365	720	0	0	0

Note: The dilution of yolk antibody is 1:300.

### REV antibody detection in serum and yolk from different SPF chicken populations

A total of 1000 yolk samples and 1000 serum samples from 10 different SPF chicken populations were detected for REV antibody. Table 3 showed that all samples tested were negative based on yolk and serum antibody detection. Our evaluation results were consistent and without false positive results, indicating that the test SPF chicken populations were not infected by REV.

**Table 3:** Detection of REV antibody from 10 SPF chicken flocks in China (random collection).

Farms No.	Age (days)	Antibody positive to REV /Total(%)	
		Yolk antibody	Sera antibody
SPF01	253	0/100 (0.00)	0/100 (0.00)
SPF02	416	0/100 (0.00)	0/100 (0.00)
SPF03	148	0/100 (0.00)	0/100 (0.00)
SPF04	170	0/100 (0.00)	0/100 (0.00)
SPF05	231	0/100 (0.00)	0/100 (0.00)
SPF06	200	0/100 (0.00)	0/100 (0.00)
SPF07	162	0/100 (0.00)	0/100 (0.00)
SPF08	340	0/100 (0.00)	0/100 (0.00)
SPF09	410	0/100 (0.00)	0/100 (0.00)
SPF10	220	0/100 (0.00)	0/100 (0.00)
**Total**		**0/1000 (0.00)**	**0/1000 (0.00)**

Note: The dilution of yolk antibody is 1:300.

## Discussion

Recently, epidemiological surveys have shown that different Chinese chicken populations are frequently infected by REV, especially in local Chinese chicken species [1–3]. To control REV infection, many measures have been employed, including the use of attenuated vaccines without REV contamination. In China and other countries, the possibility of REV contamination in attenuated poultry vaccines has been a major concern for many years. Many REV infections are thought to be caused by REV infection in contaminated attenuated vaccines, particularly for the most frequently used fowlpox virus vaccine (FPV) and anti-Marek’s Disease vaccines [5–13]. Additionally, the capability of REV to integrate into the genome of other viruses complicates its diagnosis and prevention [14–21]. Awad *et al*. detected REV in contaminated FPV vaccine using PCR identification and REV antibody detection for virus isolation and identification in vaccinated SPF chickens[7]. REV contamination in avian attenuated vaccines can lead to serious consequences, such as a significant reduction in antibody levels in vaccine-immunized chicken populations[22].

The REV contamination in attenuated vaccines may occur during the production process, but the use of REV-contaminated chicken embryos as raw materials is always the main cause. The national standards of China specify that vaccine production enterprises or SPF chicken breeding manufactures must periodically measure REV antibody levels in SPF chicken serum to evaluate the REV cleanliness in specific flocks. Because of differences in SPF chicken breeding environments, other individuals should not be allowed to enter a SPF chicken breeding area for sampling. This current approach causes both stress responses in SPF chickens and introduces the risk of false results for SPF chicken serum tests resulting from the inspection process. Therefore, the Ministry of Agriculture of China asked whether yolk antibody detection in hatching eggs could be used as a substitute for serum antibody detection to evaluate exogenous virus contamination in SPF chicken embryos.

The yolk dilution has a strong influence on the antibody detection results, as excessive high yolk concentration is prone to yield false negative or false positive results. The results of this present study showed that yolk at a 1:300 dilution gave the best goodness of fit between the antibody-negative or positive results based on yolk or serum antibody detection. To precisely and scientifically reveal the correlation between the yolk and serum antibody detection, we compared REV antibody detection results in the yolk and serum of 72 SPF chickens (40 were inoculated with REV one month prior to egg-laying) for 10 consecutive weeks. We found that for the 72 chickens, serum antibody detection results coincided with yolk antibody results at a rate of 100%. Our findings indicate that it is feasible to replace serum antibody tests with yolk antibody detection to monitor REV infection in SPF chickens.

At the optimal dilution determined in this study, a total of 1000 yolk samples and 1000 serum samples from 10 separate SPF chicken populations were tested for REV antibodies, and all showed negative results. The results of undetected antibodies showed that these chickens were not infected with REV or that although these chickens were infected with REV, not enough antibodies were detected. In order to avoid the false negative, we consider that chickens repeatedly tested negatively are not infected with REV, which is very important in flock surveillance. Additionally, detection results that used both methods were fully consistent. Importantly, no false positive results were obtained. These robust results indicate that contemporary SPF chicken embryos in China are mostly or fully not contaminated by REV. Our findings suggest that vaccine production enterprises could evaluate the REV cleanliness of SPF chicken farms by detecting antibodies in the yolk of SPF eggs. This process not only reduces the stress responses of SPF chickens during serum sampling and provides convenience for sampling, it also yields more reliable samples. Indeed, compared with serum sample results, hatching egg-based data are less prone to human error.

## Materials and methods

### REV strain

The strain REV-HA9901 was isolated in 1999 and full-length genomic sequencing had been completed (GenBank Accession No. AY842951) [23]. Supernatants of the pre-frozen virus cells at –80°C were used to calculate TCID_50_ by the Karber method; 0.1 mL supernatant of CEF cells contained 10 ^4.5^ TCID_50_.

### Rearing and virus challenge of SPF chickens

A total of 40 nineteen-week-old SPF chickens were purchased from SPAFAS Poultry Co., and were reared in HEPA-filtered negative-pressure isolators. At nineteen weeks of age, groups of 13, 14, and 13 chickens were vaccinated with 10^3^ TCID_50_ HA9901, 10^4^ TCID_50_ HA9901, and 10^5^ TCID_50_ of HA9901, respectively. All labeled chickens were separately raised within a single cage in an SPF animal feeding unit so that eggs and serum samples could corresponded 1:1 with chickens. A total of 32 SPF chickens in the same batch were reared in isolation environments as a negative control. All these chickens from each group were sacrificed by intravenous administration of barbiturates. The use of all laboratory animals in this study was approved by the scientific ethical committee of Shandong province.

### Determination of the optimal yolk dilution

The 40 inoculated SPF chickens all began laying eggs when 23-weeks-old, and the hatching eggs and serum samples were collected from each chicken. Serum samples were diluted to the optimal concentration in accordance with the instructions of the ELISA test kit for REV antibody (IDEXX Company); and yolk samples were diluted to 1:150, 1:200, 1:300, and 1:400. To minimize the possibility of human errors, paired serum and yolk from each chicken were tested using the same kit by the same laboratory staff in simultaneous ELISA experiments with identical conditions. Each sample was tested twice, and if the two values differed greatly the test was repeated. Based on these results, we determined the optimal dilution of yolk at which the detection was in accordance with that determined based on serum antibody detection.

### REV antibody detection in serum and yolk among chickens of different ages

Each week, paired egg and serum samples from each chicken were collected from 72 SPF chickens for 10 weeks from the age of 25 to 34 weeks old. If a chicken did not lay eggs on the blood-collecting day, the egg laid one day before or after the blood collection was used. For REV antibody detection, serum samples were diluted according to the manufacturer’s instructions and yolk samples were diluted in accord with the optimal dilution determined in Section 1.3. To minimize the possibility of human errors, paired serum and yolk from each chicken were tested using the same batch of kits by the same laboratory staff in simultaneous ELISA experiments with identical conditions. Each sample was tested twice, and if the two values differed greatly, tests were repeated. Finally, we compared the “goodness of fit” between the yolk antibody sampled during different stages and serum antibody measurements.

### REV antibody detection in the serum and yolk of different SPF chicken populations

Paired egg and serum samples from each chicken were sampled from 10 distinct Chinese SPF chicken populations. Serum samples were diluted in accordance with the test kit manufacturer’s instructions (IDEXX Company), and yolk samples were diluted in accordance with the optimal dilution that was determined. We separately estimated the REV cleanliness for different SPF chicken populations based on the two previously described examination methods, and compared differences in the actual operation. To minimize the introduction of human errors, paired serum and yolk samples from a chicken were tested using the same batch of kits by the same laboratory staff in simultaneous ELISA experiments with identical conditions. Each sample was tested twice, and if the two values differed greatly the tests were repeated.
